# Comparative analysis of clinical, physiological, temperamental and personality characteristics of elderly subjects and young subjects with asthma

**DOI:** 10.1371/journal.pone.0241750

**Published:** 2020-11-06

**Authors:** Michał G. Panek, Michał S. Karbownik, Piotr B. Kuna

**Affiliations:** 1 Medical University of Lodz, Department of Internal Medicine, Asthma and Allergy of the Medical University of Lodz, Lodz, Poland; 2 Medical University of Lodz, Department of Pharmacology and Toxicology of the Medical University of Lodz, Lodz, Poland; Medical University of Vienna, AUSTRIA

## Abstract

**Background:**

Asthma is a heterogeneous disease of a complex etiology in which genetic, environmental and personality variables are important factors determining the development of complicated strategies related to coping with stress and temperament traits. Our thesis is that coping styles in asthmatic patients are modified by the environment (chronic inflammation and stress) which affects individual temperament traits in the course of time. Thus, patient age is one of factors which determine the clinical image of asthma and its natural history.

**Aim:**

The aim of the study was to evaluate the variables describing stress coping styles and temperament in young (18 to 35 years old) and elderly asthmatics (aged ≥60 years).

**Material and methods:**

A total of 200 patients, 104 elderly and 96 young asthmatics were enrolled in the study. Apart from medical examination, the following tests were performed in all subjects: the Formal Characteristics of Behavior- Temperament Inventory (FCB-TI), Coping Inventory for Stressful Situations (CISS), Beck Depression Inventory, State-Trait Anxiety Inventory, and Borg Rating of Perceived Exertion (RPE) Scale.

**Results:**

Elderly patients with asthma exhibited higher intensity of anxiety as a trait, a higher level of depression and experienced dyspnea, as well as higher levels of stress coping strategies such as Avoidance-Oriented Coping (AOC), Distraction Seeking (DS) and Social Diversion (SD) compared to young asthmatics. In elderly patients, Perseverance and Sensory Sensitivity traits have been observed to decline with the duration and development of asthma at later life stages as opposed to young asthmatics, in whom these temperament characteristics are elevated.

**Conclusions:**

Asthma is a heterogeneous disease of a complex etiopathogenesis that has a complex interplay with mental health. The present study confirms a relationship between age and stress coping strategies as well as temperament traits.

## Introduction

Chronic respiratory diseases (CRD), including asthma, pose a real challenge to modern medicine, psychiatry and psychology [1–5]. Hence, there is a need to identify the mechanisms responsible for an occurrence of extrasomatic disease complications, including induction of fear, depression, adoption of different stress coping strategies and changes in the temperament. [6–11]. The above psychopathological variables are genetically conditioned at the DNA level (deoxyribonucleic acid). Via biological mediators, they affect the activation of the limbic system, which co-determines the set of personality traits (psychoticism–P, extraversion–E and neuroticism–N)–PEN theory (Eysenck’s concept of three broad traits) [12–14]. Numerous laboratory analyses confirmed an effect of many genetic determinants of CRD and inflammatory proteins in asthma on the change of the structure of the PEN traits and the taxonomy of personality. The authors revealed that the number of glucocorticoid receptors (GR) in cells as well as their function are limited in patients with mood disturbances [15–18]. GR expression is determined and regulated by the GR gene NR3C1 (nuclear receptor subfamily 3, group C, member 1 gene; glucocorticoid receptor gene) [19–21]. A relationship between the NR3C1 gene haplotype and depression and fear was also confirmed [22]. Inflammatory mediators are known to be a significant factor contributing to the development of mood disturbances in the course of inflammatory somatic diseases. Exacerbation of mood disturbances correlates with the level of many inflammatory markers: C reactive protein (CRP), IL-1, IL-2, IL-5, IL-6, IL-12, IL-13, TNF- α, Interferon-α [23–31]. It has been scientifically proven that chronic inflammation, typical for asthma, through inflammatory mediators (cytokines) secondarily reduces the CREB (Cyclic adenosine monophosphate Response Element-Binding protein) activity, the level of TRK (Tyrosine Receptors Kinases) protein and release of BDNF (Brain-Derived Neurotrophic Factor) in frontal lobes and the limbic system, which results in damage to the hippocampus and a reduced level of cerebral monoamines [23–25]. Also some drugs, such as theophylline, used in asthma therapy, contribute to an increased level of cAMP (cyclic Adenosine MonoPhosphate), but do not increase the level of the CREB protein activity, which might stimulate the limbic system and increase the release of catecholamines. This results in significant clinical changes in mood and behavior, starting with panic attacks and finishing with morbid thoughts. [32]. According to the monocyte-T-lymphocyte hypothesis, the key role in determining personality and temperament is played by the immune system via synthesis, release and uptake of monoamines in the central nervous system [25,33,34]. Decreased or increased expression of synaptic plasticity, neurogenesis, and neuromodulation via basal ganglia, the frontal cortex and the activity of the hypothalamus-pituitary axis (HPA) affect the limbic system and its activity [26,27,35–38].

Changes in the immune system, immunopathology of allergic and non-allergic asthmatic reactions, function of adrenergic and cholinergic receptors, different gene expressions, involvement of neuropeptides, as well as kinin systems are different in young and elderly asthmatics. All these factors cause anxiety disorders, which have been described in over 60% of asthma patients, and result in depressive disorders that affect 28% to 60% of these patients. The above elements induce the formation of a specific personality profile in patients, depending on age [11–16]. Some personality dimensions can play an important role in modeling the course of the disease, affecting the frequency of exacerbations, effectiveness of treatment, control of asthma signs and symptoms, quality of life, ways of coping with stress, everyday functioning in society, cooperation with a doctor and compliance with medical recommendations. The chronic nature of asthma significantly limits the physical, emotional and social functioning of patients. Temperament as a component of personality depends on biological conditions of the central nervous system and manifests itself early. Numerous genetic and physiological factors play an important role in creating temperament. According to the Moruzzi and Magoun activation theory, the biological basis of temperament may consist in the non-specific activation of the cerebral cortex by the brain stem reticular formation [12–30]. The above theses have been confirmed by a number of studies conducted with the use of electroencephalography and demonstrate that the characteristics of alpha waves and evoked potentials, which are indicators of the action potentials in the cerebral cortex, can be treated, according to Eysenck's postulates, as physiological correlates of temperament dimensions[12,13,31–35]. However, there have been no studies on the importance of temperament factors (distinguished according to the concept developed by Strelau and Zawadzki) and strategies of coping with stress in young and elderly asthmatics with regard to their psychopathological profiles, asthma severity, number of disease exacerbations and degree of its control. All these factors have a cognitive and clinical significance, as psychopathological problems are closely associated with weakening of cognitive functions, worse control of the underlying disease and intensity of its symptoms. A detailed description of the temperament characteristics as personality dimensions related to psychopathological variables would be a valuable cognitive tool enabling clinicians to take appropriate preventive actions. In this study, the authors present the phenotypes of temperament and stress coping styles in young and elderly asthmatics, which significantly differ in different age groups [1–3,10,36–39].

## Aim

The aim of this study was to compare psychopathological and personality variables (including temperament) in young and elderly patients with asthma, as well as to attempt to determine the significance and strength of these variables in the clinical picture of both disease phenotypes.

## Material and methods

### Ethical approval

The study was approved by the local Ethics Committee (Consent of the Research Review Board of the Medical University of Lodz, Łódź, Poland; no. RNN/133/09/KE). At the beginning of the study, the participants were invited to attend it voluntarily, and prior to enrolment, written informed consent was obtained from each patient. The clinical examination was performed by two investigators who were unaware of the participants' phenotypes.

Two hundred participants were included in the study (104 elderly and 96 young asthmatics). The mean age in the group of patients was 27.6 (4.9) for young asthmatics (18–35 years old) and 66.4 (5.6) years for elderly patients (aged ≥60 years) [53].

Patients with asthma were selected from asthmatics of the Department of Internal Medicine, Asthma and Allergy, the Department of Pneumology and Allergology, and the Specialist Outpatient Clinic of Pulmonary Diseases and Allergology of the Norbert Barlicki Memorial University Teaching Hospital No. 1 in Łódź, Poland by non-probability convenience sampling [9–11,19–22,40]. Posters and leaflets informing of the scientific study, approved by the Bioethics Committee of the Medical University in Lodz, were distributed all over the hospital and its outpatient clinics. Besides, the information on the study was also commonly available for Internet users (www.pta.med.pl). Potential respondents were not contacted by e-mail or text message.

The patients who had been diagnosed with chronic obstructive pulmonary disease, cardiac failure of III and IV class according to the NYHA Functional Classification, renal failure, liver insufficiency, diabetes, autoimmune diseases, neoplastic diseases, unstable angina pectoris and other severe systemic diseases were excluded from the study [9–11,19–22,40]. Functional tests of the respiratory system were conducted in compliance with the European Respiratory Society (ERS)/American Thoracic Society (ATS) guidelines. In the group of young and elderly asthmatic patients, all levels of asthma severity were assessed according to the Global Initiative for Asthma (GINA) guidelines [41]. Skin prick tests were carried out in compliance with the EAACI guidelines [42]. In order to evaluate the level of asthma control, the authors used the Asthma Control Test (ACT™), recommended by the GINA Report [43,44]. Temperament was assessed with the application of the Formal Characteristics of Behavior (Briskness, Perseverance, Sensory Sensitivity, Emotional Reactivity, Endurance, Activity) -Temperament Inventory (FCB-TI) (STRELAU) [45]. Coping Inventory for Stressful Situations (CISS: TOC—Task-Oriented Coping, EOC—Emotion-Oriented Coping, AOC—Avoidance-Oriented Coping, DS—Distraction Seeking, and SD—Social Diversion strategies) was used to evaluate the degree of coping with stress [46]. In all subjects, the authors applied the Beck Depression Inventory in a version recommended by Pużyński and Wciórka [47,48]. Trait and state anxiety were measured by the Spilberger State–Trait Anxiety Inventory (STAI), a version modified by Wrześniewski et al. and issued by the Psychological Test Laboratory of the Polish Psychological Association [49]. The level of dyspnea was measured with the use of 10-score Borg Rating of Perceived Exertion (RPE) Scale [9–11,50]. The body weight was assessed with the standard BMI Index [51–53].

### Statistical analysis

Descriptive statistics included means and standard deviations (SD) or absolute and relative frequencies, if not stated otherwise. Comparisons between young and elderly people with asthma were performed with Student’s *t*-test, Welch’s *t*-test (in case of severe violation of variance equality assumption), Mann-Whitney *U* test (in case of severe violation of normal distribution assumption and for ordinal variables), χ^2^ test (for dichotomous variables) or two-tailed Fisher's exact test (for dichotomous variables when the expected values within a cell was below five). Although not all psychometric variables were found normally distributed, they were compared between young and elderly asthma people with Student’s t-test and other general linear model analyses to allow for multivariate modeling. Each time, if possible, however, corresponding non-parametric tests were performed and they all yielded very similar results to the parametric ones. As a substantial amount of data for psychometric variables was missing, data missingness pattern was tested by comparing the means or frequencies of all other variables between the observed and missing values in each psychometric variable. Apart from complete-case analysis, missing psychometric data was imputed with multiple imputation by the chained equations technique to perform sensitivity analysis of psychometric data. Additionally, the analyses adjusted to some covariates were performed. The false discovery rate for all the comparisons was controlled at the level of 0.05 with the Benjamini and Hochberg correction for testing multiple hypotheses. P-values below 0.05 were considered statistically significant. The analysis was performed using STATISTICA 13.1 software (StatSoft, Tulsa, OK, USA) and R Software version 3.6.1 (R Core Team 2019).

## Results

It is worth pointing out that at least 80% of the asthma patients invited to participate in the scientific study responded positively to the invitation. Table 1 presents the detailed characteristics of the asthmatics study groups. Moreover, this Table contains the data on medications taken by patients, comorbidities and other important clinical parameters (e.g. smoking).

**Table 1:** Detailed characteristics and description of young (18–35 years old) and elderly asthmatic patients (aged ≥60 years).

Variable	Number (frequency) or mean (standard deviation)^1^		*P*-value for comparison of young *vs* elderly subjects with asthma
	Young subjects with asthma (n = 96)	Elderly subjects with asthma (n = 104)	
**Sex**			
Male	49 (51.0%)	28 (26.9%)	**0.0005**^**5**^
Female	47 (49.0%)	76 (73.1%)	
**Age** [years]	27.6 (4.9)	66.4 (5.6)	**<0.0001**^**6**^
**BMI** [kg/m^2^]	24.7 (4.5)	28.4 (4.8)	**<0.0001**^**7**^
**Allergy**			
Seasonal	59 (61.5%)	24 (23.3%)^2^	**<0.0001**^**5**^
Perennial	64 (66.7%)	28 (27.2%)^2^	**<0.0001**^**5**^
Number of allergens	median: 3, 1^st^-3^rd^ quartile: 1–5, range: 0–20	median: 0, 1^st^-3^rd^ quartile: 0–1, range: 0–11	**<0.0001**^**6**^
**Smoking**			
Current smokers	17 (17.7%)	7 (6.7%)	**0.017**^**5**^
Ex-smokers	14 (14.6%)	47 (45.2%)	**<0.0001**^**5**^
Number of pack years	1.4 (3.5), median: 0, 1^st^-3^rd^ quartile: 0–1, range: 0–20	9.0 (13.9)^3^, median: 0.23, 1^st^-3^rd^ quartile: 0–15, range: 0–80	**<0.0001**^**6**^
**Rhinitis**			
Rhinitis	83 (86.5%)	34 (32.7%)	**<0.0001**^**5**^
nGCs* rhinitis treatment	39 (47.0%)	9 (26.5%)	0.041^5^
Episodic rhinitis	10 (10.4%)	4 (3.8%)	0.069^5^
Chronic rhinitis	73 (76.0%)	30 (28.8%)	**<0.0001**^**5**^
Seasonal rhinitis	30 (31.3%)	12 (11.5%)	**0.0006**^**5**^
Perennial rhinitis	53 (55.2%)	22 (21.2%)	**<0.0001**^**5**^
**Medications**			
Anti-H_1_ treatment	60 (62.5%)	32 (30.8%)	**<0.0001**^**5**^
Proton pump inhibitor treatment	10 (10.4%)	24 (23.1%)	**0.017**^**5**^
Anti-H_2_ treatment	3 (3.1%)	5 (4.8%)	0.72^8^
Medications hypersensitivity	21 (21.9%)	23 (22.1%)	0.97^5^
**Comorbidities**			
Nasal polyps	2 (2.1%)	9 (8.7%)	0.042^5^
Neurological or neurosurgical diseases	13 (13.5%)	22 (21.2%)	0.16^5^
Lipid disorders	4 (4.2%)	20 (19.2%)	**0.0011**^**5**^
Thyroid goiter	2 (2.1%)	5 (4.8%)	3 (1.3%)
Hypothyroidism	2 (2.1%)	11 (10.6%)	10 (4.5%)
Hyperthyroidism	1 (1.0%)	9 (8.7%)	12 (5.4%)
Atherosclerosis	0 (0.0%)	7 (7.1%)^4^	**0.014**^**8**^
Hypertension	3 (3.1%)	52 (52.5%)^4^	**<0.0001**^**5**^
Arrhythmia	2 (2.1%)	13 (13.1%)^4^	**0.0038**^**5**^
Coronary heart disease	1 (1.0%)	19 (19.2%)^4^	**<0.0001**^**5**^
Myocardial infarction	0 (0.0%)	5 (5.1%)^4^	0.059^8^
Other cardio-vascular diseases	1 (1.0%)	14 (14.1%)^4^	**0.0006**^**5**^
Chronic obstructive pulmonary disease	8 (8.3%)	27 (26.2%)^2^	**0.0009**^**5**^
Other pulmonary diseases*	1 (1.0%)	4 (3.9%)^2^	0.37^8^
Peptic ulcer disease	2 (2.1%)	11 (10.6%)	**0.015**^**5**^
Duodenal ulcer disease	1 (1.0%)	3 (2.9%)	0.62^8^
Gastro-esophageal reflux disease	8 (8.3%)	35 (33.7%)	**<0.0001**^**5**^
Neoplastic diseases	0 (0.0%)	13 (12.5%)	**0.0003**^**5**^
Immunodeficiency	2 (2.1%)	2 (1.9%)	1.0^8^
Allergen-specific immunotherapy	13 (13.5%)	2 (1.9%)	**0.0018**^**5**^

^1^ if not stated otherwise

^2^ one case with a missing value

^3^ two cases with a missing value

^4^ five cases with a missing value

^5^ Pearson's χ2 test

^6^ Mann-Whitney U test

^7^ Student’s t-test

^8^ Fisher’s exact test

*Nasal Glucocorticosteroids

**including sarcoidosis, bronchiectasia and tuberculosis; Benjamini-Hochberg corrected significance level: 0.037; p-values marked in bold indicate statistically significant difference; The author’s own analysis.

The prevalence of mild asthma was 29.2% (28 patients) in young patients and 24.0% (25) in the elderly. The prevalence of moderate, severe steroid-sensitive and severe steroid-resistant asthma was 51.0% (49), 19.8% (19), and 0.0% (0), respectively, in young asthmatics and 40.4% (42). 34.6% (36), and 1.0% (1) in elderly asthmatics. The studied populations appeared to be different with regards to the time of the diagnosis of the disease (early-onset asthma vs. late-onset asthma) and not only with regards to asthma duration only (since its diagnosis–duration in years according to age ranges). However, no significant differences in the dose of administered glucocorticosteroids (GCS) were observed. The group of elder asthmatics was characterized by a significantly higher level of complications associated with GCS treatment [28 (26.9%) *vs* 12 (12.6%)]. The analyzed groups of patients did not differ in the intensity of basic treatment [systemic GCS, inhaled GCS, long-acting beta 2-agonists (LABA), long-acting muscarinic antagonists (LAMA), anti-leukotriene agents (anti-LT) and short-acting muscarinic antagonists (SAMA)]. However, the higher level of daily intake of short-acting beta-2-agonists (SABA) in elderly asthmatics [18 (18.2%) *vs* 6 (6.6%)] and the more frequent use of methylxanthines [16 (15.4%) *vs* 4 (4.2%)] were recorded. Young and elderly asthmatics did not differ in the frequency of severe asthma exacerbations. Both examined populations statistically significantly differed in the results of spirometry parameters [FEV1 L (forced expiratory volume in 1 second, liter), FEV1% (percentage) predicted, FVC L (forced vital capacity, liter), FVC% predicted and FEV1% FVC%] and the level of asthma control (ACT—asthma control test): 17.4 (5.2) *vs* 19.7 (5.1). The detailed results regarding asthma severity according to the GINA report, the medications used, treatment intensity, drug complications, lung function parameters and asthma control levels according to ACT^TM^ are presented in Table 2.

**Table 2:** Detailed characteristics of asthma severity, levels and frequency of using asthma medications and results of spirometry parameters in young (18–35 years old) and elderly asthmatics (aged ≥60 years).

Variable	Number (frequency) or mean (standard deviation)^1^		*P*-value for the comparisons
	Young subjects with asthma (n = 96)	Elderly subjects with asthma (n = 104)	
Mild asthma	28 (29.2%)	25 (24.0%)	0.042^2^
Moderate asthma	49 (51.0%)	42 (40.4%)	
Severe asthma	19 (19.8%)	36 (34.6%)	
Severe-steroid resistant asthma	0 (0.0%)	1 (1.0%)	
Asthma diagnosis time < 3 years	12 (12.5%)	0 (0.0%)	**<0.0001**^**2**^
Asthma diagnosis time 3–7 years	9 (9.4%)	3 (3.0%)	
Asthma diagnosis time 7–16 years	18 (18.7%)	3 (3.0%)	
Asthma diagnosis time 16–40 years	57 (59.4%)	25 (24.7%)	
Asthma diagnosis time > 40 years	0 (0.0%)	70 (69.3%)	
Medications			
No iGCS	17 (17.9%)	15 (14.4%)	0.17^2^
iGCs <1000μg	54 (56.8%)	53 (51.0%)	
iGCs≤1000μg	24 (25.3%)	36 (34.6%)	
Systemic GCs	20 (21.1%)	26 (25.0%)	0.51^3^
GCs side effects	12 (12.6%)	28 (26.9%)	**0.012**^3^
Asthma exacerbations	12 (12.6%)	20 (19.4%)	0.19^3^
LABA	63 (66.3%)	80 (77.7%)	0.075^3^
0x/week SABA	30 (33.0%)	30 (30.3%)	**0.033**^**2**^
≤1x/2weeks SABA	39 (42.8%)	25 (25.2%)	
>1x/2weeks to <2x/week SABA	4 (4.4%)	6 (6.1%)	
≥2x/week to <7x/week SABA	7 (7.7%)	11 (11.1%)	
≥7x/week SABA	5 (5.5%)	9 (9.1%)	
≥1x/day SABA	6 (6.6%)	18 (18.2%)	
0x/week SAMA	88 (91.7%)	91 (87.5%)	0.27^2^
≤1x/2weeks SAMA	4 (4.2%)	1 (1.0%)	
>1x/2weeks to <2x/week SAMA	2 (2.1%)	2 (1.9%)	
≥2x/week to <7x/week SAMA	0 (0.0%)	0 (0.0%)	
≥7x/week SABA	1 (1.0%)	1 (1.0%)	
≥1x/day SAMA	1 (1.0%)	9 (8.6%)	
LAMA	5 (5.2%)	11 (10.6%)	0.16^**3**^
aLT	29 (30.2%)	32 (30.8%)	0.93^**3**^
MTX	4 (4.2%)	16 (15.4%)	**0.0082**^**3**^
FEV1(L)	3.16 (0.80)	1.75 (0.68)	**<0.0001**^4^
FEV1(%) pred.	82.6 (18.1)	73.5 (22.3)	**0.0019**^4^
FVC(L)	4.44 (1.01)	2.65 (0.83)	**<0.0001**^4^
FVC(%) pred.	98.8 (13.6)	91.3 (21.3)	**0.0035**^**5**^
FEV1%FVC(%)	71.0 (11.1)	65.4 (11.6)	**0.0007**^4^
FEV1%FVC(%) pred.	84.3 (12.6)	84.5 (15.0)	0.92^4^
ACT^TM^	19.7 (5.1), median: 21, 1^st^-3^rd^ quartile: 16–24	17.4 (5.2), median: 18, 1^st^-3^rd^ quartile: 13–22	**0.0011**^**2**^

^1^ if not stated otherwise

^2^ Mann-Whitney U test

^3^ Pearson's χ2 test

^4^ Student’s t-test

^5^ Welch’s t-test; List of abbreviations: GCs-glucocorticoids, iGCs-inhaled GCs, SABA-Short-Acting Beta2-Agonists, SAMA-Short-Acting Muscarinic Antagonist, LABA-Long-Acting Beta2-Agonists, LAMA-Long-Acting Muscarinic Antagonist, aLT-anti-leukotrienes, MTX-Methylxanthines, pred-predicted value, ACT-Asthma Control Test^TM^; Benjamini-Hochberg corrected significance level: 0.039; p-values marked in bold indicate statistically significant difference; The author’s own analysis.

Psychopathological variables were analyzed in detail. In the population of elderly asthmatics, higher levels of depression (Beck Scale) [12.7 (11.1–14.3) *vs* 6.2 (4.6–7.8)] and anxiety as a trait in the STAI X2 questionnaire [44.6 (42.8–46.5) *vs* 39.0 (37.1–41.0)], as well as a higher level of perceived dyspnea (Borg Scale) [3.51 (3.03–3.99) *vs* 2.02 (1.53–2.51)] were found. Importantly, the differences were stable across various sensitivity analyses. Anxiety as a state (STAI X1 questionnaire) did not differentiate the groups of young and elderly asthmatics. The studied populations differed in two strategies of coping with stress: AOC [48.8 (45.6–52.0) *vs* 42.0 (37.4–46.6); p = 0.0198] and DS [22.0 (20.2–23.9) *vs* 17.3 (14.6–19.9); p = 0.0047], however, the difference in AOC did not remain significant in imputed dataset. Of all six temperament traits, Perseverance turned out to be higher in young asthmatics than in the elderly [13.7 (11.8–15.7) *vs* 10.8 (9.5–12.1)], but also only in complete-case analyses. The intensity of sensory sensitivity was statistically significantly higher in younger than elderly asthmatics [16.6 (15.1–18.0) vs 13.6 (12.6–4.6)]. The remaining temperament traits did not differ between the two groups. Table 3 presents a detailed analysis of the variables of Beck, STAI, Borg, stress coping strategies and temperament traits.

**Table 3:** Detailed description of analysis, of temperament components and stress coping strategies in the studied population of young and elderly asthmatics.

Variable	Raw analysis			Adjusted analysis*		
	Mean (95% CI)		P-value	Mean (95% CI)		P-value
	Young subjects with asthma	Elderly subjects with asthma		Young subjects with asthma	Elderly subjects with asthma	
Beck	**6.2 (4.6–7.8)**	**12.7 (11.1–14.3)**	**<0.0001**	**7.0 (5.3–8.6)**	**11.4 (9.8–13.0)**	**0.0005**
Beckimp	6.2(4.6–7.8)	12.5(11.0–14.0)	<0.0001	6.7(5.1–8.3)	11.5(10.0–13.1)	0.0001
STAI X1	36.1 (33.9–38.3)	38.8 (36.7–41.0)	0.076	37.0 (34.5–39.5)	38.2 (35.8–40.5)	0.54
STAIX1imp	36.1(33.9–38.2)	38.9(36.8–41.0)	0.062	36.8(34.4–39.2)	38.1(35.8–40.4)	0.47
STAI X2	**39.0 (37.1–41.0)**	**44.6 (42.8–46.5)**	**<0.0001**	**39.6 (37.4–41.7)**	**43.6 (41.6–45.7)**	**0.014**
STAIX2imp	39.0(37.1–40.9)	44.3(42.5–46.1)	<0.0001	39.6(37.5–41.6)	43.4(41.4–45.4)	0.016
Borg	**2.02 (1.53–2.51)**	**3.51 (3.03–3.99)**	**<0.0001**	**2.20 (1.67–2.73)**	**3.43 (2.91–3.94)**	**0.0028**
Borgimp	2.02(1.53–2.51)	3.55(3.08–4.02)	<0.0001	2.25(1.72–2.77)	3.44(2.94–3.95)	0.0032
TOC	59.1 (54.9–63.4)	57.6 (54.7–60.6)	0.57	57.3 (52.0–63.0)	59.7 (56.0–63.4)	0.52
TOCimp	57.9(56.3–59.5)	57.0(55.4–58.5)	0.39	56.9(55.2–58.6)	58.3(56.7–60.0)	0.28
EOC	42.7 (37.7–47.6)	45.9 (42.5–49.4)	0.28	44.1 (39.0–49.2)	45.2 (41.7–48.7)	0.75
EOCimp	43.7(41.8–45.5)	45.5(43.8–47.3)	0.14	43.9(42.0–45.8)	45.1(43.2–46.9)	0.43
AOC	**42.0 (37.4–46.6)**	**48.8 (45.6–52.0)**	**0.0198**	**38.8 (33.4–44.2)**	**50.5 (46.8–54.2)**	**0.0035**
AOCimp	45.3(43.3–47.3)	45.6(43.7–47.5)	0.81	45.5(43.3–47.8)	45.6(43.4–47.7)	0.97
DS	**17.3 (14.6–19.9)**	**22.0 (20.2–23.9)**	**0.0047**	**16.5 (13.4–19.6)**	**22.5 (20.4–24.6)**	**0.0076**
DSimp	19.1(18.0–20.1)	21.5(20.5–22.4)	0.0008	19.1(18.0–20.3)	21.2(20.2–22.3)	0.016
SD	16.7 (14.6–18.7)	18.5 (17.0–19.9)	0.16	**15.0 (12.4–17.6)**	**19.3 (17.5–21.1)**	**0.01996**
SDimp	18.0(17.3–18.7)	17.9(17.2–18.6)	0.79	17.7(16.9–18.6)	17.8(17.0–18.6)	0.92
Briskness	15.7 (13.5–18.0)	12.5 (11.0–14.0)	0.022	15.7 (12.9–18.4)	12.6 (10.7–14.4)	0.11
Brisknessimp	13.6(12.7–14.4)	12.7(11.8–13.5)	0.14	13.3(12.4–14.3)	13.1(12.2–14.0)	0.75
Perseverance	**13.7 (11.8–15.7)**	**10.8 (9.5–12.1)**	**0.016**	**14.4 (12.0–16.9)**	**10.1 (8.4–11.8)**	**0.016**
Perseveranceimp	11.9(11.2–12.6)	11.2(10.5–11.9)	0.15	11.8(11.0–12.6)	11.2(10.5–12.0)	0.40
Sensory sensitivity	**16.6 (15.1–18.0)**	**13.6 (12.6–14.6)**	**0.0015**	**17.6 (15.5–19.7)**	**13.1 (11.6–14.5)**	**0.0040**
Sensorysensitivityimp	15.6(15.0–16.2)	14.3(13.7–14.9)	0.0031	15.5(14.9–16.2)	14.4(13.8–15.1)	0.028
Emotional Reactivity	11.9 (9.5–14.3)	11.0 (9.3–12.6)	0.51	12.3 (9.0–15.6)	10.6 (8.3–12.8)	0.45
EmotionalReactivityimp	11.8(11.0–12.6)	11.4(10.6–12.2)	0.56	11.8(10.9–12.6)	11.3(10.5–12.1)	0.46
Endurance	7.0 (4.4–9.6)	6.9 (5.1–8.7)	0.95	6.5 (2.9–10.2)	7.2 (4.8–9.7)	0.77
Enduranceimp	7.8(6.8–8.8)	7.1(6.1–8.1)	0.33	7.3(6.2–8.4)	7.6(6.6–8.7)	0.67
Activity	9.1 (6.8–11.5)	7.4 (5.8–9.0)	0.23	7.7 (4.5–10.8)	8.2 (6.1–10.4)	0.79
Activityimp	9.6(8.7–10.4)	8.4(7.5–9.2)	0.056	9.4(8.5–10.4)	8.7(7.7–9.6)	0.32

CI-confidence intervals. Benjamini-Hochberg corrected significance level 0.020. Results in bold are statistically significant. Results in gray are based on imputed data.

*adjusted for: sex, BMI, current smoking, FVC%, number of allergens, exacerbations, frequency of the use of SABA, systemic GCS, inhaled GCS up to 1000ug/d and >1000 ug/d. Abbreviations: CISS–strategies of coping with stress, (TOC—Task-Oriented Coping; EOC—Emotion-Oriented Coping; AOC—Avoidance-Oriented Coping; DS—distraction seeking; SD–social diversion). FCB–TI–temperamental components (Briskness, Perseverance, Sensory Sensitivity, Emotional Reactivity, Endurance, Activity). Detailed data presented in the text. The author’s own analysis.

The missing data in the analyzed database constituted 10.8% of all the spreadsheet cells. Psychometric data was missing in 58.3% of cells, whereas the remaining data was missing in 0.636% of cells. Psychometric data was missing in the following number of asthmatic patients: 5.0% (10 patients) for the Borg questionnaire, 5.5% (11 patients) for the Beck Depression Inventory and STAI, 77.0% (154 patients) for CISS and 78.0% (156 patients) for STRELAU.

Missing data resulted to some extent from unwillingness of the patient to fill in particular questionnaire and a burden associated with a lot of time consumed.

Further analysis of the missingness pattern revealed that, apart from data on the use of proton pump inhibitors, there was no strong evidence indicating that the data was not missing completely at random (for detailed results see S1 Table). Consequently, the abovementioned complete-case analyses could be fairly justifiable.

## Discussion

The article presents a unique study which analyses psychopathological variables in young and elderly asthmatics. Scientific literature reports that the age when young asthmatics become elderly asthmatics is not clearly determined [53–60]. This fact makes it difficult to provide unequivocal definitions. The problem was more detailed in the Limitations section. Despite these limitations, we believe that our results provide a valuable contribution to various dimensions of asthma in young and elderly subjects.

In this discussion, we cannot ignore the fact that there was a disproportion between sex, age and BMI among the studied populations. Young asthmatics also had more frequent allergies as well as sensitivity to seasonal and perennial allergens, while the number of allergens sensitizing elderly asthmatics was statistically significantly lower (see Table 1). Furthermore, elderly asthmatics smoked more cigarettes for a longer period of time, which obviously resulted in significantly lower spirometry parameters, thus affecting the feeling of dyspnea. Allergic rhinitis (86.5%) was the most frequent comorbidity among young asthmatics. Whereas, elderly asthmatics more often suffered from hypertension and other cardio-vascular diseases, including coronary heart disease (19.2%). It should be highlighted that the above comorbidities and other clinical conditions presented in Table 1 are factors significantly affecting the feeling of perceived dyspnea and the level of disease control.

Another problem is the recognition of chronic, persistent bronchial obstruction in asthmatics which leads to the diagnosis of chronic obstructive pulmonary disease (COPD) in some of them basing on spirometric but not clinical parameters: [27 (26.2%) elderly asthmatics *vs* 8 (8.3%) young asthmatics]. Taking the above data into account, as well as the disproportion in age and duration of asthma, it should be noted that both populations did not differ significantly in terms of the intensity of anti-inflammatory treatment of asthma according to GINA, which is presented in Table 2. Nevertheless, elderly asthmatics took more emergency rescue medications than young patients, which could explain their worse asthma control and lower spirometric parameters (see Table 2). Elderly patients were more often treated with systemic GCS (similarly result) and more often had complications after systemic therapy (statistically significant result). Researchers' attention was drawn to the fact that elderly patients significantly more often used methylxanthines [16 (15.4%) *vs* 4 (4.2%)], which are not recommended in primary asthma therapy. They are drugs burdened with a very narrow therapeutic index and may induce various complications, including cardiac ones.

To overcome the potential confounding effect of some analyzed covariates, raw analyses were enriched with adjusted sensitivity analyses. The adjusted analysis was additionally applied to compare and verify our results, which are presented in the Table 3. After these analyses, we found that some disproportions resulting from sex, BMI, current smoking, FVC%, number of allergens, exacerbations, intensity of using SABA, systemic steroids, inhaled steroids up to 1000ug/d, and above 1000ug/d in the studied groups do not affect the results of psychometric analysis [52].

Elderly asthmatics were characterized by a significantly higher level of anxiety as a trait, level of perceived dyspnea and depression, as well as higher levels of three stress coping strategies (AOC, DS, SD) as compared to young asthmatics (see Table 3). These results appeared to depend on age rather than any covariates. Higher levels of anxiety as a trait can be explained in elderly patients by their anticipation of danger from outside (inhalation of allergens leading to an asthmatic attack) or inside the body (exacerbation of non-infectious asthma in the course of the natural history of the disease), which is usually accompanied by additional symptoms of mental, motor and vegetative stimulation. Unlike fear, this anticipation is an internal process which is not associated with imminent danger but accompanied by cognitive perceptions of experiencing danger. In evolutionary terms, anxiety as a threat signal enabled people to adapt to changes in the environment and motivated them to take protective measures allowing to survive [61]. In our study, young asthmatics had lower levels of anxiety, dyspnea and depression. This can be explained by the fact that the occurrence of frequent exacerbations in young patients induced a better adaptation to a strong stressor (episode of dyspnea) than in elderly asthmatics, thus the severity of psychopathological variables was lower in young asthmatics than in other patients who did not have exacerbations in the past year or experienced them very rarely. Earlier studies of the authors on depression and anxiety in patients with asthma showed that they experience more severe depression and anxiety as compared to healthy subjects [9,10,30,45,52]. Asthma as a chronic disease reduces the rate of changes in patients’ behavior in response to the changing conditions of surroundings, environment and disease progression. Hence, more severe episodes of dyspnea and depression in elderly asthma patients may require a change in behavioral strategy and the way of seeking help. Three styles of coping with stress, Avoidance-Oriented Coping (AOC), Distraction Seeking (DS) and Social Diversion Strategies (SD), were dominant in elderly asthma patients. The AOC style is the least effective of all stress coping strategies because it is based on distracting one's attention from the current state. This results in a lack of confrontation with emerging difficulties and failure to acquire problem-solving abilities. The person using this strategy believes that the stressful situation will be spontaneously resolved. We distinguish two sub-styles in the AOC style: DS (e.g. sleeping) and SD. With increasing age, the number of people declaring this way of coping with stressful situations increases (up to 31% in the 55–79 age group), which has been also confirmed by our studies [52,62,63]. In the literature, one can also find data indicating that the style focused on avoidance is associated with the patients’ body weight and increases with BMI index. This strategy also correlated with allergy and its value was definitely lower in atopic patients, but it increased with the occurrence of large variability of symptoms, i.e. exacerbation of allergy-related diseases. Obese patients with symptom attacks may reject thoughts of excessive weight and disease to a greater degree than patients with lower BMI and perennial symptoms, the former trying to repress and avoid experiencing their health problems. Nevertheless, they were intensively involved in displacement activities, which positively correlated with patients’ age, duration of asthma, and body weight expressed by the BMI index [9,10,30,45,52,62,63]. The AOC style grew in patients with higher values of anxiety as a trait and was associated with taking inhaled medications (systemic GCS and LABA) [52]. The value of the style of engaging in displacement activities was higher in older patients, perhaps due to the greater number of medications and higher doses of anti-asthmatic agents taken, which improved the patients’ quality of life and increased their ability to undertake various activities of daily living, such as cleaning, sleeping, watching television, seeking friends or establishing new social relationships (see Table 3) [45,52].

Temperament is the most inherited structural component of all personality factors. Higher levels of personality traits such as Perseverance and Sensory Sensitivity in young asthmatics may result from a short duration of asthma, or may be a constitutional risk factor for asthma. Our research does not provide the answers to these questions, but both variants seem to be possible [9,10,30,45,52]. Perseverance has been defined as a "tendency to continue or repeat behaviors and experience emotional states after cessation of the stimulus (situation) that induced these behaviors (states)" which seems to reflect the psychophysical states of young asthmatics after an episode of dyspnea. These are frequent situations to which the patients are in some way used to and are able to deal with them [11–16]. Moreover, literature data indicates the similar situations, as Perseverance has been found to decline with the age at asthma onset and duration of the disease. The value of this factor grew with the intensity of anxiety as a trait. Reduced Perseverance in asthmatics, which is a temporary trait of behavior, can also be a response to chronic inflammation and chronic symptoms [12–15,30,45,52]. Sensory Sensitivity (ease of arousing emotions in the case of poor stimulation) was definitely higher in young asthmatics. In the study, Sensory Sensitivity was observed to decrease with the age of patients and time of disease onset, which might be caused by the reduced ability of the patients to respond to sensory stimuli of low value due to the patients’ reduced excitability and sensitivity. Scientific investigations reveal that this dimension grew in patients along with the intensity of depression and anxiety as a state and trait. This may be explained by weakening of the tendency to spontaneous and immediate reaction to environmental and emotional stimuli [12–15,30,45,52].

Available studies on the relationship of psychopathological variables, including coping stress strategies and temperament traits, and stressors to immunological assays as well as a meta-analytic review do not provide information on the relationship between these processes and the age of patients (young asthmatics *vs* elderly asthmatics). This fact makes an analysis of the results considerably more difficult. In general, it should be concluded that obstructive disorders are associated with considerable emotional burden of the patient, which may result in the development of mood disturbances and anxiety disorders. However, the literature data are contradictory, and do not take into account psychopathological changes related to the age of the patients, the diagnosis of the disease and its duration [1,2,5,9–16,30,45,52].

The Discussion should also mention the problem of missing data, which may be a limitation of the study. Scientific studies indicate that even a considerable lack of data allows for proper interpretation of results of statistical analyses unless the lack is MNAR (missing not at random) [64]. According to our analysis, missing completely at random pattern of data missingness was likely. However, missing not at random (MNAR) pattern could not be also ruled out. MNAR pattern is not to be experimentally determined in a particular dataset and it may result from some sociodemographic and psychological features of respondents [https://bmcmedresmethodol.biomedcentral.com/articles/10.1186/s12874-020-01038-3]. Consequently, the present results, particularly for the variables with many missing, may be somewhat biased [64]. In order to evaluate potential bias resulting from missing observations of psychometric data, one should not consider the amount of missingness. It was proposed that missing as much as 90% of data may contribute to no bias (Madley-Dowd et al. 2019) [64]. The pattern of missingness, however, plays a crucial role in evaluating potential bias. As presented in Suppl. Table there is little evidence linking any observed covariate with missingness. Age of the subjects may be to some extent linked to missingness pattern of CISS and STRELAU if Benjamini-Hiochberg correction is to be neglected. If this association is true-positive and missingness in these variables are not at random (which cannot be experimentally assessed), some bias may result performing complete-case analysis or an analysis following multiple imputation. On the other hand, this is unlikely that age links to missingness in Borg, Beck and STAI variables (S1 Table). In this case, even if not at random missingness pattern in these variables is observed, this may cancel out due to similar amount of missingness in young and elderly asthmatics (if only no interaction of an age and missingness pattern occurs), and no substantial bias is expected for Borg, Beck and STAI as resulting from data missingness. This consideration was appreciated in the discussion section and pointed as a limitation of our study [64].

### Limitations

Professional literature reports that various age ranges for elderly asthmatics may make an analysis of data difficult, particularly when we compare the data with those obtained by other authors, who adopted different age ranges for patients. The applied age limit of 60 years may be considered questionable because the American Thoracic Society Workshop Report regards subjects over 65 years of age as people advanced in years. However, because the processes associated with ageing become more significant after the age of 60 and because this age cut-off has been used in previous studies, we believe that the age of 60 years is important as a lower age limit for elderly people. Nonetheless, there is a number of literature reports in which the age of 70 years seems to be appropriate cut-off for considering a patient to be elderly [53,57–60]. Finally, the patients with asthma were recruited from the reference care centre, which may limit the ability to generalize outcomes of our study. Moreover, it is possible that asthma control may be more problematic in elderly patients treated in primary health care institutions by general practitioners.

A certain limitation of the study might be high missing data, which may considerably alter its results. However, it should be stressed there was substantial amount of missingness for some psychometric data, particularly CISS and STRELAU. This resulted from: a lack of consent of the patient to fill in such a questionnaire, insufficient time provided to give a lot of answers to the questions, the patients’ unwillingness to fill in such questionnaires, particularly psychological ones (the patient’s decision). However, a further analysis of the missingness pattern revealed that, apart from data on the use of proton pump inhibitors, there was no strong evidence against the missing completely at random pattern (for detailed results see S1 Table). Consequently, the abovementioned complete-case analyses are fairly justifiable [64].

Lack of other investigations on stress coping strategies and temperament components carried out in groups of similar number of young and elderly asthmatics limits our study and potential interpretation as well as comparison of the findings. The drawn conclusions are probably not definitive and also have some limitations. The above research should be continued on greater samples of patients with more representativeness. Therefore, our results should be treated as preliminary, but they significantly add new cognitive values ​​to the Regulatory Theory of Temperament [12–15,52]. Based on the available research, our theses seem to be crucial in understanding the complex etiopathogenesis of asthma and personality variables (depression, anxiety, stress coping strategies, and temperament traits according to the Regulatory Theory of Temperament), however, the variables we present might not be the only factors affecting the processes described in the article [12–15,52].

Summing up, in the present study, we have confirmed the theses on the impact of individual factors (age) on stress coping styles and relationships with temperament traits (according to the Regulatory Theory of Temperament) in patients suffering from asthma being the disease of a complex etiopathogenesis that has a complex interplay with mental health. We have found significant differences between young and elderly asthmatics and have shown that elderly patients are characterized by higher levels of anxiety as a trait, and higher levels of depression and perceived dyspnea. The elderly patients also exhibit higher levels of stress coping strategies, such as AOC, DS, and SD, as compared to young asthmatics. Furthermore, we have found that with the duration of asthma and its later development Perseverance and Sensory Sensitivity traits are different in elderly asthma patients as opposed to young asthmatics in whom these traits are higher. The above investigations should be continued in multi-centre scientific research including pathway analysis which could explain the correlates observed. While analyzing the literature data, it should be stated that genetic, individual and environmental factors significantly affect the development of asthma, and formation of stress coping styles as well as temperament factors which are components of personality.

## Supporting information

S1 Table Supplementary table presenting and comparing missing vs non-missing cases for each covariate. (DOCX)

## References

[1] Panek M, Witusik A, Pietras T, Kuna P (2010): Psychiatric disorders in patients with bronchial asthma—doctrine and therapy. *TERAPIA* 4: 69–72.

[2] Grabowska P, Targowski T, Niedziałkowski P (2006): Psychological problems in patients with bronchial asthma. *Alergologia współczesna* 2: 14–16.

[3] Świtaj P (2008): Experience of social stigma and discrimination in patients diagnosed with schizophrenia. Warszawa: Wydawnictwo Instytutu Psychiatrii i Neurologii 10.1097/IMI.0b013e31816755c3

[4] Sęk H (2001): Introduction to Clinical Psychology. Warszawa: Wydawnictwo Naukowe SCHOLATR.

[5] Stoudemire A (1985): Psychosomatic theory and pulmonary disease. Asthma as a paradigm for the biopsychosocial approach. *Adv Psychosom Med*.14: 1–15. 4072802

[6] Haverkamp F, Staab D, Müller-Sinik K, Rünger M (2004): Methodological perspectives for the assessment of adaptation in chronic diseases, exemplified by asthma bronchiale in childhood. *Klin Padiatr* 216: 1–6. 10.1055/s-2004-817681 14747963

[7] Vink NM, Boezen HM, Postma DS, Rosmalen JG (2013): Basal or stress-induced cortisol and asthma development: the TRAILS study. *Eur Respir J* 41: 846–852. 10.1183/09031936.00021212 22790914

[8] Razvodovsky YE (2010): Psychosocial distress as a risk factor of asthma mortality. *Psychiatr Danub* 22: 167–172. 20562741

[9] Pietras T, Panek M, Witusik A, Wujcik R, Szemraj J, Górski P, et al (2011): Analysis of the correlation between level of anxiety, intensity of depression and bronchial asthma control. *Post Dermatol Alergol* 28: 15–22.

[10] Pietras T, Panek M, Witusik A, Wujcik R, Szemraj J, Górski P, et al (2010): Analysis of the correlation between anxiety, depression, intensity of dyspnoea and severity of the bronchial asthma disease process. *Post Dermatol Alergol* 27: 390–399.

[11] Pietras T, Witusik A, Panek M, Hołub M, Gałecki P, Wujcik R, et al (2009): Anxiety and depression in patients with obstructive diseases. *Pol Merkur Lekarski* 26: 631–635. 19711730

[12] Strelau J (2006): Temperament as a regulator of behavior in fifty-year research perspective (in Polish). Gdańsk: Gdansk Psychology Publishing Company.

[13] Strelau J (2014): Individual differences–history–determinants–applications (in Polish). Warszawa: Scholar Publishing House.

[14] Strelau J, Zawadzki B, Oniszczenko W, Sobolewski A, Bieniek A (2004): Temperament and stress coping styles as the moderators of symptoms of posttraumatic stress due to a disaster. In: Strelau J(Ed.). *Personality and extreme stress* (in Polish). Gdańsk: Gdansk Psychology Publishing Company.

[15] Zawadzki B, Popiel A (2012): Temperamental Traits and Severity of PTSD Symptoms Data From Longitudinal Studies of Motor Vehicle Accident Survivors. *J Ind Diff* 33: 257–267.

[16] Pompili M, Innamorati M, Gonda X, Erbuto D, Forte A, Ricci F, et al (2014): Characterization of patients with mood disorders for their prevalent temperament and level of hopelessness. *J Affect Disord* 166: 285–291. 10.1016/j.jad.2014.05.018 25012443

[17] Ritsner M, Susser E (2004): Temperament types are associated with weak self-construct, elevated distress and emotion-oriented coping in schizophrenia: evidence for a complex vulnerability marker? *Psychiatry Res* 128: 219–228. 10.1016/j.psychres.2004.06.007 15541778

[18] Pariante CM, Miller AH (2001): Glucocorticoid receptors in major depression: relevance to pathophysiology and treatment. *Biol Psychiatry* 49: 391–404. 10.1016/s0006-3223(00)01088-x 11274650

[19] Panek M, Pietras T, Antczak A, Fabijan A, Przemęcka M, Górski P, et al (2012): The N363S and I559N single nucleotide polymorphisms of the h-GR/NR3C1 gene in patients with bronchial asthma. *Int J Mol Med* 30: 142–150. 10.3892/ijmm.2012.956 22469783

[20] Panek M, Pietras T, Antczak A, Górski P, Kuna P, Szemraj J (2012): The role of functional single nucleotide polymorphisms of the human glucocorticoid receptor gene NR3C1 in Polish patients with bronchial asthma. *Mol Biol Rep* 39: 4749–4757. 22015776 10.1007/s11033-011-1267-3 PMC3294211

[21] Panek M, Pietras T, Fabijan A, Miłanowski M, Wieteska L, Górski P, et al (2013): Effect of glucocorticoid receptor gene polymorphisms on asthma phenotypes. *Exp Ther Med* 5: 572–580. 10.3892/etm.2012.809 23407653 PMC3570225

[22] Panek M, Pietras T, Szemraj J, Kuna P (2014): Association analysis of the glucocorticoid receptor gene (NR3C1) haplotypes (ER22/23EK, N363S, BclI) with mood and anxiety disorders in patients with asthma. *Exp Ther Med* 8: 662–670. 10.3892/etm.2014.1734 25009637 PMC4079411

[23] Margaretten M, Julian L, Katz P, Yelin E (2011): Depression in patients with rheumatoid arthritis: description, causes and mechanisms. *Int J Clin Rheumtol* 6: 617–623. 10.2217/IJR.11.6 22211138 PMC3247620

[24] Marques AH, Silverman MN, Sternberg EM (2009): Glucocorticoid dysregulations and their clinical correlates. From receptors to therapeutics. *Ann N Y Acad Sci* 1179: 1–18. 10.1111/j.1749-6632.2009.04987.x 19906229 PMC2933142

[25] Elomaa AP, Niskanen L, Herzig KH, Viinamäki H, Hintikka J, Koivumaa-Honkanen H, et al (2012): Valkonen-Korhonen M, Harvima IT, Lehto SM. Elevated levels of serum IL-5 are associated with an increased likelihood of major depressive disorder. *BMC Psychiatry* 12: 2 10.1186/1471-244X-12-2 22230487 PMC3266629

[26] Pace TWW, Hu F, Miller AH (2007): Cytokine-effects on glucocorticoid receptor function: Relevance to glucocorticoid resistance and the pathophysiology and treatment of major depression. *Brain Behav Immun* 21: 9–19. 10.1016/j.bbi.2006.08.009 17070667 PMC1820632

[27] Irwin MR, Miller AH (2007): Depressive disorders and immunity: 20 years of progress and discovery. *Brain Behav Immun* 21:374–383. 10.1016/j.bbi.2007.01.010 17360153

[28] Khairova RA, Machado-Vieira R, Du J, Manji HK (2009): A potential role for pro-inflammatory cytokines in regulating synaptic plasticity in major depressive disorder. *Int J Neuropsychopharmacol* 12: 561–578. 10.1017/S1461145709009924 19224657 PMC2771334

[29] Myint AM, Leonard BE, Steinbusch HW, Kim YK (2005): Th1, Th2, and Th3 cytokine alterations in major depression. *J Affect Disord* 88: 167–173. 10.1016/j.jad.2005.07.008 16126278

[30] Zorrilla EP, Luborsky L, McKay JR, Rosenthal R, Houldin A, Tax A, et al (2001): The relationship of depression and stressors to immunological assays: A meta-analytic review. *Brain Behav Immun* 15: 199–226. 10.1006/brbi.2000.0597 11566046

[31] Raison CL, Capuron L, Miller Ah(2006): Cytokines sing the blues: Inflammation of the pathogenesis of major depression. *Trend Immunol* 27: 24–31. 10.1016/j.it.2005.11.006 PMC3392963 16316783

[32] Favreau H, Bacon SL, Joseph M, Labrecque M, Lavoie KL (2012): Association between asthma medications and suicidal ideation in adult asthmatics. *Respir Med* 106: 933–941. 10.1016/j.rmed.2011.10.023 22495109

[33] Maes M, Smith R, Scharpe S (1995): The monocyte-T-lymphocyte hypothesis of major depression. *Psychoneuroendocrinology* 20: 111–116. 10.1016/0306-4530(94)00066-j 7899532

[34] Nadjar A, Bluthé RM, May MJ, Dantzer R, Parnet P (2005): Inactivation of the cerebral NFkappaB pathway inhibits interleukin-1beta-induced sickness behavior and c-Fos expression in various brain nuclei. *Neuropsychopharmacology* 30: 1492–1499. 10.1038/sj.npp.1300755 15900319

[35] Capuron L, Pagnoni G, Demetrashvili MF, Lawson DH, Fornwalt FB, Woolwine B, et al (2007): Nemeroff CB, Miller AH. Basal ganglia hypermetabolism and symptoms of fatigue during interferon-alpha therapy. *Neuropsychopharmacology* 32: 2384–2392. 17327884 10.1038/sj.npp.1301362

[36] Capuron L, Pagnoni G, Demetrashvili M, Woolwine BJ, Nemeroff CB, Berns GS, et al (2005): Anterior cingulate activation and error processing during interferon-alpha treatment. *Biol Psychiatry* 58: 190–196. 10.1016/j.biopsych.2005.03.033 16084839 PMC1366492

[37] McAfoose J, Baune BT (2009): Evidence for a cytokine model of cognitive function. *Neurosci Biobehav Rev* 33: 355–366. 10.1016/j.neubiorev.2008.10.005 18996146

[38] Nelson T, Ur C, Gruol D (2002): Chronic interleukin-6 exposure alters electrophysiological properties and calcium signaling in developing cerebellar purkinje neurons in culture. *J Neurophysiol* 88: 475–486. 10.1152/jn.2002.88.1.475 12091569

[39] Fasmer OB, Halmøy A, Eagan TM, Oedegaard KJ, Haavik J (2011): Adult attention deficit hyperactivity disorder is associated with asthma. *BMC Psychiatry* 11:128 10.1186/1471-244X-11-128 21819624 PMC3163523

[40] Panek M, Pietras T, Fabijan A, Zioło J, Wieteska L, Małachowska B, et al (2014): Identification and association of the single nucleotide polymorphisms, C-509T, C+466T and T+869C, of the TGF-β1 gene in patients with asthma and their influence on the mRNA expression level of TGF-β1. *Int J Mol Med* 34: 975–986. 10.3892/ijmm.2014.1894 25119113 PMC4152139

[41] Miller MR, Hankinson J, Brusasco V, Burgos F, Casaburi R, Coates A, Crapo R, et al (2005): ATS/ERS Task Force. Standardisation of spirometry. *Eur Respir J* 26: 319–338. 10.1183/09031936.05.00034805 16055882

[42] Dreborg S, Backman A, Bosomba A (1989): Skin tests used for type I Allergy testing. Position paper, EAACI subcommittee. *Allergy* 44: 1–59.

[43] Nathan R, Sorkness C, Kosinski M, Schatz M, Li J, Marcus P, et al (2004): Development of the Asthma Control Test: A survey for assessing asthma control. *J Allergy Clin Immunol* 113: 59–65. 14713908 10.1016/j.jaci.2003.09.008

[44] http://www.ginasthma.org/.

[45] Zawadzki B, Strelau J (1997): Formal Characteristics of Behavior–Temperament Inventory (FCB-TI). Manual (in Polish). Warszawa: Psychological Test Laboratory of the Polish Psychological Association.

[46] Strelau J, Jaworowska A, Wrześniewski K, Szczepaniak P (2013): Coping in Stress Situations (CISS) Questionnaire. Manual. Third Edition (in Polish). Warszawa: Psychological Test Laboratory of the Polish Psychological Association.

[47] Beck A, Ward C, Mendelson M, Mock J, Erbaugh J (1961): An inventory for measuring depression. *Arch Gen Psychiatry* 4: 561–571. 10.1001/archpsyc.1961.01710120031004 13688369

[48] Pużyński S, Wciórka J (2002): Narzędzia oceny stanu psychicznego. In: Psychiatria Bilikiewicz A, Pużyński S, Rybakowski J i wsp. Wrocław: Wydawnictwo Medyczne Urban & Partner, pp. 453–538.

[49] Wrześniewski K, Sosnowski T, Matusik D (2002): Inwentarz Stanu i Cechy Lęku STAI. Polska Adaptacja STAI. Podręcznik. Warszawa: Pracownia Testów Psychologicznych Polskiego Towarzystwa Psychologicznego.

[50] Grammatopoulou E, Skordilis E, Koutsouki D, Baltopoulos G (2008): An 18-item standardized Asthma Quality of Life Questionnaire-AQLQ(S). *Qual Life Res* 17: 323–32. 10.1007/s11136-007-9297-y 18080214

[51] Global Database on Body Mass Index. World Health Organization (2006): BMI Classification Retrieved.

[52] Panek M, Pietras T, Witusik A, Wieteska Ł, Małachowska B, Mokros Ł, et al Identification and association of relationships between selected personal and environmental factors and formal components of temperament and strategies of coping with stress in asthmatic patients. Physiol Behav. 2015 10 1;149:269–78. 10.1016/j.physbeh.2015.06.023 26079811

[53] Boulet L, Robitaille C, Deschesnes F, Villeneuve H, Boulay M. Comparative Clinical, Physiological, and Inflammatory Characteristics of Elderly Subjects With or Without Asthma and Young Subjects With Asthma. Chest. 2017 12;152(6):1203–1213. 10.1016/j.chest.2017.09.019 28941741

[54] Czyż P, Furgał M, Nowobilski R, de Barbaro B, Pulka G. The significance of selected psychopathological and personality variables in the course of allergic and non-allergic asthma. Psychiatr. Pol. 2014; 48(5):1047–1058. 10.12740/pp/25802 25639023

[55] Murtagh E, Heaney L, Gingles J. Prevalence of obstructive lung disease in a general population sample: the NICECOPD study. Eur J Epidemiol. 2005;20(5):443–453. 10.1007/s10654-005-1248-8 16080593

[56] McHugh MK, Symanski E, Pompeii LA. Prevalence of asthma among adult females and males in the United States: results from the National Health and Nutrition Examination Survey (NHANES), 2001–2004. J Asthma. 2009;46(8):759–766. 10.1080/02770900903067895 19863277 PMC11494460

[57] Busse PJ, Birmingham JM, Calatroni A. The effect of aging on sputum inflammation and asthma control. J Allergy Clin Immunol. 2017;139(6):1808–1818. 10.1016/j.jaci.2016.09.015 27725186 PMC5385166

[58] Brooks CR, Gibson PG, Douwes J. Relationship between airway neutrophilia and ageing in asthmatics and nonasthmatics. Respirology. 2013;18(5):857–865. 10.1111/resp.12079 23490307

[59] Skloot GS, Busse PJ, Braman SS. An Official American Thoracic Society Workshop Report: Evaluation and Management of Asthma in the Elderly. Ann Am Thorac Soc. 2016;13(11):2064–2077. 10.1513/AnnalsATS.201608-658ST 27831798 PMC5466180

[60] Busse PJ, Mathur SK. Age-related changes in immune function: effect on airway inflammation. J Allergy Clin Immunol. 2010;126(4):690–699. 10.1016/j.jaci.2010.08.011 20920759 PMC3297963

[61] Mielimąka M, Rutkowski K, Cyranka K, Sobański J, Dembińska E, Müldner-Nieckowski Ł. Trait and state anxiety in patients treated with intensive short-term group psychotherapy for neurotic and personality disorders. Psychiatr. Pol. ONLINE FIRST Nr 36. 10.12740/PP/OnlineFirst/60537. 29432511

[62] Huber L. Style adaptacyjne do sytuacji stresowych w różnych grupach wiekowych a choroby cywilizacyjne XXI wieku. Probl Hig Epidemiol 2010; 91(2): 268−275.

[63] Kurpas D, Kusz J, Jedynak T, Mroczek B. Preferable styles of coping with stress among the chronically ill patients. Family Medicine & Primary Care Review 2012, 14, 3: 393–395.

[64] Madley-Dowd P, Hughes R, Tilling K, Heron J. The proportion of missing data should not be used to guide decisions on multiple imputation. J Clin Epidemiol 2019 6;110:63–73. 10.1016/j.jclinepi.2019.02.016 30878639 PMC6547017

