# Supplementary material for: Comparative analysis of clinical, physiological, temperamental and personality characteristics of elderly subjects and young subjects with asthma
Source: PLoS One. 2020 Nov 6;15(11):e0241750. doi: 10.1371/journal.pone.0241750 (PMC7647458; doi:10.1371/journal.pone.0241750)
Supplement: S1 Table — (DOCX) [file pone.0241750.s001.docx]

Supplementary Table

| Covariate | *P*-value for comparison of missing *vs* non-missing cases for each covariate | | | |
| --- | --- | --- | --- | --- |
|  | Beck, STAI | Borg | CISS subscales | STRELAU subscales |
| Asthma severity  (mild, moderate, severe, severe-steroid resistant) | 0.93 | 0.73 | 0.36 | 0.24 |
| Age (years) | 0.86 | 0.60 | 0.015 | 0.015 |
| BMI | 0.45 | 0.60 | 0.045 | 0.036 |
| Number of allergens | 0.28 | 0.52 | 0.19 | 0.23 |
| Asthma diagnosis time | 0.45 | 0.14 | 0.11 | 0.15 |
| Inhaled GCS (<1000µg or ≥1000µg) | 0.49 | 0.52 | 0.16 | 0.14 |
| Frequency of administration of inhaled SABA drugs | 0.36 | *0.78* | 0.16 | 0.12 |
| Frequency of administration of inhaled SMA drugs | 0.31 | 0.33 | 0.0076 | 0.0048 |
| Number of pack years | 0.76 | 0.36 | 0.025 | 0.014 |
| FEV1(L) | 0.30 | 0.33 | 0.089 | 0.07 |
| FEV1(%) pred. | 0.19 | 0.65 | 0.46 | 0.22 |
| FVC(L) | 0.26 | 0.25 | 0.19 | 0.27 |
| FVC(%) pred. | 0.10 | 0.53 | 0.57 | 0.49 |
| FEV1%FVC(%) | 0.82 | 0.60 | 0.31 | 0.11 |
| FEV1%FVC(%) pred. | 0.71 | 0.38 | 0.51 | 0.22 |
| ACT^TM^ | 0.75 | 0.67 | 0.22 | 0.16 |
| Sex: female - 0, male - 1; | 0.43 | 0.22 | 0.66 | 0.47 |
| Seasonal allergy | 0.10 | 0.44 | 0.20 | 0.17 |
| Perennial allergy | 0.50 | 0.69 | 0.78 | 0.76 |
| Systemic GCS | 0.31 | 0.95 | 0.18 | 0.12 |
| LABA | 0.51 | 0.38 | 0.19 | 0.26 |
| SABA | 0.30 | 0.55 | 0.76 | 0.94 |
| LAMA | 0.31 | 0.34 | 0.67 | 0.74 |
| a-LT | 0.27 | 0.97 | 0.070 | 0.039 |
| MTX | 0.92 | 1.0 | 0.74 | 0.82 |
| GCs complications | 0.99 | 0.87 | 0.016 | 0.0087 |
| Hypersensitivity to medications | 0.66 | 0.53 | 0.65 | 0.78 |
| Rhinitis | 0.13 | 0.58 | 0.018 | 0.020 |
| nGCS rhinitis treatment | 0.80 | 0.66 | 0.11 | 0.065 |
| Anti-H_1_ | 0.20 | 0.70 | 0.29 | 0.27 |
| Gastro-esophageal reflux disease | 0.78 | 0.91 | 0.20 | 0.14 |
| Proton pump inhibitors | 0.12 | 0.55 | **0.0003** | **0.0001** |
| Anti-H_2_ | 0.38 | 0.32 | 0.89 | 0.83 |
| Allergen-specific immunotherapy | 0.84 | 0.76 | 0.35 | 0.40 |
| Asthma exacerbations | 0.85 | 0.74 | 0.43 | 0.34 |
| Nasal polyps | 0.59 | 0.52 | 0.73 | 0.66 |
| Neurological or neurosurgical diseases | 0.45 | 0.83 | 0.39 | 0.30 |
| Lipid disorders | 0.52 | 0.42 | 0.79 | 0.88 |
| Thyroid goiter | 0.30 | 0.25 | 0.72 | 0.67 |
| Hypothyroidism | 0.37 | 0.39 | 0.17 | 0.14 |
| Hyperthyroidism | 0.52 | 0.46 | 0.82 | 0.88 |
| Atherosclerosis | 0.0074 | 0.0042 | 0.75 | 0.70 |
| Hypertension | 0.54 | 0.39 | 0.26 | 0.17 |
| Arrhythmia | 0.18 | 0.13 | 0.025 | 0.030 |
| Coronary heart disease | 0.055 | 0.0015 | 0.48 | 0.40 |
| Myocardial infarction | 0.0007* | 0.0003* | 0.38 | 0.34 |
| Other cardio-vascular diseases | 0.18 | 0.13 | 0.77 | 0.69 |
| Chronic obstructive pulmonary disease | 0.11 | 0.13 | 0.4 | 0.31 |
| Other pulmonary diseases (including sarcoidosis, bronchiectasia, tuberculosis) | 0.58 | 0.60 | 0.36 | 0.33 |
| Peptic ulcer disease | 0.72 | 0.076 | 0.17 | 0.14 |
| Duodenal ulcer disease | 0.084 | 0.064 | 0.19 | 0.17 |
| Neoplasmatic diseases  present or in history | 0.37 | 0.39 | 0.040 | 0.14 |
| Immunodeficiency | 0.084 | 0.064 | 0.27 | 0.28 |
| Episodic rhinitis | 0.35 | 0.37 | 0.14 | 0.16 |
| Chronic rhinitis | 0.30 | 0.92 | 0.11 | 0.11 |
| Seasonal rhinitis | 0.81 | 0.47 | 0.27 | 0.35 |
| Perennial rhinitis | 0.17 | 0.24 | 0.14 | 0.11 |
| Ex-smoker | 0.27 | 0.17 | 0.47 | 0.34 |
| Current smoker | 0.76 | 0.84 | 0.44 | 0.37 |

Supplementary Table presenting and comparing missing *vs* non-missing cases for each covariate

List of abbreviations: GCs-glucocorticoids, iGCs-inhaled GCs, SABA-Short-Acting Beta2-Agonists, SAMA-Short-Acting Muscarinic Antagonist, LABA-Long-Acting Beta2-Agonists, LAMA-Long-Acting Muscarinic Antagonist, aLT-anti-leukotrienes, MTX-Methylxanthines, pred-predicted value, ACT-Asthma Control Test^TM^; Benjamini-Hochberg corrected significance level: 0.00084. *As the expected values within some cells of contingency tables were far below five, Fisher exact test was more suitable in these cases. This test yielded non-significant results at the Benjamini-Hochberg corrected significance level, namely *p*=0.027 and *p*=0.022 for Beck-STAI and Borg, respectively. *P*-values marked in bold indicate statistically significant difference. The author’s own analysis.
